# AL101, a gamma-secretase inhibitor, has potent antitumor activity against adenoid cystic carcinoma with activated NOTCH signaling

**DOI:** 10.1038/s41419-022-05133-9

**Published:** 2022-08-05

**Authors:** Renata Ferrarotto, Vasudha Mishra, Elad Herz, Adar Yaacov, Oz Solomon, Rami Rauch, Adi Mondshine, Maria Motin, Tal Leibovich-Rivkin, Matti Davis, Joel Kaye, Christopher R. Weber, Le Shen, Alexander T. Pearson, Ari J. Rosenberg, Xiangying Chen, Alka Singh, Jon C. Aster, Nishant Agrawal, Evgeny Izumchenko

**Affiliations:** 1 Department of Head and Neck Medical Oncology, The University of Texas MD Anderson Cancer Center, Houston, TX USA; 2 Department of Medicine, Section of Hematology and Oncology, University of Chicago, Chicago, IL USA; 3 Ayala Pharmaceuticals, Rehovot, Israel; 4 Department of Pathology, University of Chicago, Chicago, IL USA; 5 Department of Pathology, Brigham and Women’s Hospital and Harvard Medical School, Boston, MA USA; 6 Department of Surgery, Section of Otolaryngology-Head and Neck Surgery, University of Chicago, Chicago, IL USA

**Keywords:** Head and neck cancer, Targeted therapies

## Abstract

Adenoid cystic carcinoma (ACC) is an aggressive salivary gland malignancy with limited treatment options for recurrent or metastatic disease. Due to chemotherapy resistance and lack of targeted therapeutic approaches, current treatment options for the localized disease are limited to surgery and radiation, which fails to prevent locoregional recurrences and distant metastases in over 50% of patients. Approximately 20% of patients with ACC carry NOTCH-activating mutations that are associated with a distinct phenotype, aggressive disease, and poor prognosis. Given the role of NOTCH signaling in regulating tumor cell behavior, NOTCH inhibitors represent an attractive potential therapeutic strategy for this subset of ACC. AL101 (osugacestat) is a potent γ-secretase inhibitor that prevents activation of all four NOTCH receptors. While this investigational new drug has demonstrated antineoplastic activity in several preclinical cancer models and in patients with advanced solid malignancies, we are the first to study the therapeutic benefit of AL101 in ACC. Here, we describe the antitumor activity of AL101 using ACC cell lines, organoids, and patient-derived xenograft models. Specifically, we find that AL101 has potent antitumor effects in in vitro and in vivo models of ACC with activating *NOTCH1* mutations and constitutively upregulated NOTCH signaling pathway, providing a strong rationale for evaluation of AL101 in clinical trials for patients with NOTCH-driven relapsed/refractory ACC.

## Introduction

Adenoid cystic carcinoma (ACC) is a relatively uncommon secretory gland malignancy with a high propensity for perineural invasion, locoregional recurrence, and distant metastasis despite curative-intent treatment. Due to its insidious infiltrative growth pattern, ACC is often advanced by the time of clinical recognition. The therapeutic management for patients with locoregional and recurrent/metastatic ACC is limited to surgery and radiation, as no systemic agent has been found to be effective in improving long-term disease control [1]. The mortality rate of ACC remains high, with over 60% of the patients succumbing to the disease within 15 years of diagnosis. Therefore, new therapeutic approaches for treating ACC are urgently needed.

Like other malignancies, ACC is thought to arise through genetic and epigenetic aberrations that lead to the mis-expression of tumor suppressor genes and oncogenes. Molecular and genetic characterization of ACC has begun to reveal the most common driver mutations in this still incompletely understood cancer [2–5]. One common driver mutation in ACC consists of chromosomal rearrangements that produce *MYB-NFIB* (~70% of the tumors) or *MYBL1-NFIB* fusion genes, which appear to have a central role in the genesis of ACC. While representing useful markers in the diagnosis of ACC, MYB family members, like other transcription factors, remain difficult therapeutic targets, and the prognostic and biologic significance of MYB fusion genes are uncertain [6]. Other driver mutations in ACC involve genes that encode epigenetic regulators (e.g., MLL2, MLL3, EP300, SMARCA2, SMARCC1, CREBBP, and KDM6A), pro-growth factors (e.g., FGFR2, PIK3CA, MYC, KRAS, BRAF), and DNA damage/checkpoint regulators (ATM, CDKN2A, TP53) [2–5, 7]. Recurrent copy number losses (e.g., of chromosome 1p36) and amplifications (e.g., of chromosome 7p14.1 and 14q11.2) have also been noted [2–5]. However, the fraction of ACC with mutations in these genes that present opportunities for targeted therapy is small.

By contrast, recently emerging data suggest that the NOTCH pathway is a tractable, rational therapeutic target in ACC [7, 8]. The NOTCH gene family contains four paralogs encoding large type 1 transmembrane receptor signaling proteins, NOTCH1, 2, 3, and 4. Normal NOTCH signaling is initiated by binding the receptor to a ligand (JAG1, JAG2, DLL1, or DLL4) on an adjacent cell. This event elicits conformational changes in the extracellular domain of NOTCH that makes it susceptible to successive cleavages by ADAM10 and γ-secretase. The latter cleavage releases the NOTCH intracellular domain (NICD), which migrates to the nucleus and forms a transcription complex that upregulates the expression of downstream target genes. These upregulated genes, in turn, regulate diverse cellular functions in a context-specific fashion, many of which have the potential to influence the behavior of cancers through both cell autonomous and non-autonomous mechanisms [9–13].

Particular cancers are marked by the presence of NOTCH-activating mutations, which either disrupt the extracellular NOTCH negative regulatory region (NRR), leading to ligand-independent proteolysis and NICD generation, or a C-terminal PEST domain, leading to stabilization of NICD [9]. Among these cancers is ACC, in which activating NOTCH mutations are found in ~20% of tumors [7, 8, 14, 15]. According to the largest analysis (*n* = 1045) conducted across several retrospective sequencing datasets [8], these mutations predominantly occur in *NOTCH1*, although mutations in *NOTCH2*, *NOTCH3*, and *NOTCH4* have also been identified [2, 5, 7, 8]. A retrospective analysis of three independent cohorts demonstrated that ACCs with *NOTCH1* activating mutations are associated with a poorer median overall survival and progress four times faster than those patients without activating mutations [8, 15, 16]. The first retrospective study of 102 ACCs revealed that tumors with *NOTCH1* mutations are associated with solid histology, advanced-stage disease at presentation, liver and bone metastases, and shorter relapse-free (12.5 vs 33.9 months) and median overall survival (29.6 vs 121.9 months) compared to tumors with wild-type (WT) *NOTCH1* [15]. The second retrospective study in 84 patients with recurrent/metastatic ACC found that median overall survival was significantly shorter in those with *NOTCH1*-mutated tumors compared to *NOTCH1* WT malignancies (55.1 vs 204.5 months) [8]. Furthermore, among *NOTCH1* mutant tumors, activating mutations were associated with significantly poorer survival (31.1 vs 73.8 months) [8]. These findings were recently supported in an independent cohort in which activating *NOTCH1* mutations were again associated with poorer overall survival (48 vs 195.6 months) [16]. Recurrent mutations in genes encoding proteins that regulate the activity of the NOTCH transcription complex, such as *SPEN* and *FBXW7*, have also been identified in ACC genomes, further implicating the NOTCH pathway in ACC tumorigenesis [2, 7, 15].

Based on the proposed multifaceted pro-oncogenic roles of NOTCH signaling in cancer [17–20], several γ-secretase inhibitors (GSIs) have been developed [15, 21–26] and tested in preclinical studies [27–32] and Phase I/II trials in patients with advanced solid tumors, including ACC [15, 33], either as a single agent or in combination with targeted therapeutics or chemotherapy [33]. However, the overall response rates to NOTCH-targeted therapy have been suboptimal. Factors limiting success to date include use of GSIs with suboptimal pharmacokinetic (PK)/pharmacodynamic properties, dose-limiting toxicity, and the failure to use biomarkers of NOTCH activation as selection criteria for clinical trial entry [33–36]. Thus, the potential of NOTCH inhibitors such as GSIs has yet to be fully explored.

AL101 is an investigational small molecule GSI that potently inhibits all four NOTCH paralogs and prevents the upregulation of NOTCH target genes [22]. While a number of recent preclinical studies have shown robust AL101 antitumor activity in several in vivo cancer models [22, 37], a comprehensive evaluation of its effect in ACC is lacking, in part due to the scarcity of experimental model systems for ACC.

Here we demonstrate that AL101 monotherapy inhibits the proliferation of NOTCH-activated ACC organoids at nanomolar concentrations. Furthermore, AL101 is well tolerated in vivo and has significant antitumor effects in ACC patient-derived xenograft (PDX) models with *NOTCH1* activating mutations, but not in PDX models with WT *NOTCH* genes. Taken together, our results provide a strong foundation for the clinical development of AL101 as a targeted monotherapy in patients with NOTCH-activated ACC.

## Methods

### Cell lines and reagents

The human ACC cell line HACC2A was received from Dr Jacques Nȍr (University of Michigan), the UFH2 cell line was received from Dr Frederic Kaye (University of Florida), and the ACC52 cell line was received from Dr Lurdes Quiemado (University of Oklahoma). Cells were monitored for mycoplasma using the MycoDetect kit (Greiner Bio-One). HACC2A cells were cultured in DMEM medium (Gibco) supplemented with 10% FBS, 200 mM L-glutamine, antibiotic-antimycotic (100X) (Gibco), 400 ng/ml hydrocortisone, 20 ng/ml epidermal growth factor, 5 µg/ml insulin and bovine brain extract (Lonza). UFH2 cells were cultured in DMEM+GlutaMAX medium (Gibco) supplemented with 10% FBS and 5000 U/ml penicillin-streptomycin (Gibco 15070063). ACC52 cells were cultured in RPMI 1640 medium (Corning) supplemented with 10% FBS, antibiotic-antimycotic (100X), 20 ng/ml epidermal growth factor, 400 ng/ml hydrocortisone, and 5 μg/ml insulin. AL101 (batch: 3H66027) was obtained from Ayala Pharmaceuticals. All other chemicals used in this study were purchased from Sigma and prepared according to the manufacturer’s recommendations.

### Organoid preparation

Freshly obtained surgical samples were digested first with a mixture of collagenase and dispase, followed by TrypLE (ThermoFisher). After passing through a mesh, cells were embedded in Matrigel (Corning) and cultured in four different organoid media formulations in parallel. These formulations have the same base media containing the growth factors EGF, Noggin (Sigma), R-spondin (Sigma), and FGF10 (ThermoFisher) as well as N-acetylcysteine (ThermoFisher), nicotinamide, Y-27632 (Rho kinase inhibitor), A83-01 (TGF-β signaling inhibitor), N2, and B27 (all from Sigma). Additional components of these media include forskolin (adenylyl cyclase activator), CHIR99021 (Wnt activator), gastrin I, prostaglandin E2, FGF2, hydrocortisone, and heregulin β1 (all from Sigma). The cells that demonstrated the most robust growth were further expanded, and fractions of cells were harvested for histology, DNA and RNA isolation, or cryopreserved at low passage numbers.

### IHC staining

Sections (4-micron) prepared from FFPE PDX tumors were stained for NOTCH1 intracellular domain (NICD1) (Cell Signaling, 4147S, 1:200), MYC (Abcam, clone Y69, catalog ab32072; 0.56 μg/ml), or Ki67 (BioCare, clone SP6, catalog CRM326, 1:100) using a Bond III automated immunostainer (Leica). Staining was carried out in the Dana Farber/Harvard Cancer Center Specialized Histopathology Core Laboratory, which is certified by the College of American Pathologists and meets Clinical Laboratory Improvement Amendments standards. Staining was developed using the Bond Polymer Refine Detection Kit (Leica). Slides were counterstained with hematoxylin and reviewed by a board-certified anatomic pathologist (JCA).

### Cell viability assays

For cell lines, relative viability was determined using an Alamar Blue assay as outlined by the manufacturer (AbDSerotec). New media containing 1/10 volume of Alamar Blue reagent was added to the wells and cells were incubated at 37 °C for 1 h. Fluorescence (560 nm excitation, 590 nm emission wavelengths) was measured using a SpectraMax-Plus384 fluorometer (Sunnyvale). Percent viability was determined by comparing DMSO treatment to inhibitor treatment. For organoids, ATP measurements (CellTiter-Glo Luminescent Cell Viability Assays, Promega) were used to assess proliferation and viability. Medium was discarded and CellTiter-Glo 3D reagent was added to each well. After incubating 30 min at room temperature on a rotary shaker, bioluminescence activity was assessed using a plate luminometer.

### Reverse transcription and real-time PCR

RNA was reverse transcribed to cDNA using Superscript III (Invitrogen) and then used as a template for real-time PCR. Gene amplification was carried out on a StepOnePlus system (Applied Biosystems) using TaqMan Gene Expression Assays (Applied Biosystems). Assay IDs were: ACTB-Hs01060665_g1, HEY1-Hs00232618_m1. HEY2-Hs01012057_m1, NRARP-Hs04183811_s1, MYC-Hs00153408_m1, and HES5-Hs01387463_g1. All reactions were performed in triplicate and relative RNA quantity was calculated after normalizing to ACTB expression by the 2^−ΔΔCT^ method.

### Xenograft models

Early passage PDX tissues were obtained through the Adenoid Cystic Carcinoma Research Foundation. All animal procedures were performed at XenoSTART (South Texas Accelerated Research Therapeutics, San Antonio) following Institutional Animal Care and Use Committee protocols. Fragments of tumor (~70 mm^3^) were implanted subcutaneously into the flanks of 6–12-week-old female nu/nu athymic nude mice (The Jackson Laboratories or Charles River Laboratories). Upon reaching 150–300 mm^3^ tumor volume, mice were randomized to either treatment (*n* = 5) or vehicle (*n* = 5–10) groups using blinded block randomization and therapeutic dosing was implemented. Tumor dimensions were measured using digital calipers blinded to the treatment group and tumor volume was calculated using the formula: TV = width^2^ × length × 0.52. Tumors were harvested at termination, weighted, and used for histology, immunohistochemical staining, and RNA sequencing (RNA-seq) analysis (*n* = 3–5 per arm). Percent mean tumor growth inhibition (%TGI) induced by AL101 was calculated relative to the untreated control group using the formula: %TGI = 1 – (AL101 final – AL101 baseline) / (Control final – Control baseline).

### NOTCH luciferase reporter assay

Full-length (FL) human *NOTCH1 (FL-NOTCH1)*, *ΔECD-NOTCH1*, *ACCx9-I1680N*, and *ACCx11-S1723ins28* transcripts were synthesized by GenScript and ligated into the pcDNA3.1 (+FLAG) expression vector. Constructs were transiently transfected into U2OS cells using Lipofectamine 2000 reagent (Invitrogen, #11668019) and assessed for their ability to activate a NOTCH-sensitive luciferase reporter gene [38, 39]. U2OS cells were used because of their transfectability and low basal NOTCH activity. Briefly, cells were co-transfected (in five biological replicates) with 10 ng of pcDNA3.1 expression construct, a NOTCH-sensitive firefly luciferase reporter gene (TP1-CSLx12-FF), and an internal control Renilla luciferase plasmid (pRL-TK, Promega). Normalized firefly luciferase activities were measured in whole cell extracts prepared 48 h after transfection using the Dual-Luciferase kit (Promega #E1960) and an Infinite M200 luminometer (Tecan).

### RNA sequencing

RNA-seq was performed on an Illumina NovaSeq-6000 instrument. Adapters were trimmed with Cutadapt [40]. For PDXs, mouse reads were filtered out using an approach described by Callari et al. [41]. Human reads were aligned to human reference genome GRCh37/hg19 using STAR and STAR-fusion [42, 43]. Gene expression levels were calculated using featureCounts [44] and gene expression levels were normalized using DESeq2 [45]. Differentially expressed (DE) genes were detected using DESeq2 according to the following parameters: (i) average gene expression >50 normalized reads, (ii) log2(fold-change) >1 or log2(fold-change) <−1, (iii) false discovery rate (FDR) < 0.05.

### Pathway enrichment analysis

Pathway enrichment analysis was performed using minimal hyper-geometric statistics [46], based on an approach similar to that described by Eden et al. [47]. MSigDB c2 (curated gene sets: Kyoto Encyclopedia of Genes and Genomes (KEGG)/Reactome pathways) [48] were used as pathway references. Multiple hypothesis correction of the *p* values was performed by controlling for FDR [49].

### NOTCH target gene signature and gene expression clustering analysis

Known NOTCH target genes were curated from the literature and used to create a 21-gene signature that co-clustered ACC and triple-negative breast cancer (TNBC) tumors [50] that were responsive to GSIs, including specimens harboring NOTCH gain of function alterations such as single-nucleotide variants, indels or fusions [51, 52]. Hierarchical clustering analysis was done based on the Euclidean Distance method (Ward.D2 linkage) and cluster selection was done using SigClust2 [53]. A signature of 480 NOTCH-related genes was collected from (1) PathwaysCommons (the gene-gene interaction table was downloaded, filtered out, and only cases where NOTCH genes regulate the expression of the second gene, were retained (interaction type: “controls-expression-of”)); (2) KEGG [54]; (3) PID [55]; (4) MSigDB (https://www.gsea-msigdb.org/gsea/msigdb/collections.jsp); and (5) the 21 direct NOTCH target genes described above.

### NOTCH activation caller

The NOTCH activation caller is a method relying on two algorithms: single sample Gene Set Enrichment Analysis (ssGSEA) [48, 56]; and k–Top Scoring Pairs (kTSP), a ranking-based classification algorithm that selects gene pairs whose expression levels switch their ranking between the two classes of interest [57–59]. For ssGSEA, a GSVA R package [60] was used with the set of 21 NOTCH-related genes described above. For each sample, a higher score indicates greater enrichment for NOTCH target genes. For kTSP, the in-house scripts were used on a set of 21 NOTCH-related genes, whose expression is expected to be higher in NOTCH-activated tumors, and a pre-defined set of four genes (*LXN*, *RAPGEF3*, *TMEM154*, and *LGR6*) whose expression is expected to be lower in NOTCH-activated tumors (derived from analyzing downregulated genes in TNBC and ACC PDX models as well as TNBC [50] and chronic lymphocytic leukemia [61] cell lines with activating NOTCH mutations curated from the literature). Briefly, the kTSP algorithm scores each sample based on the proportion of how many gene pairs comply with the expected rule of: “down” < “up”. Tumors were judged as being “NOTCH activated” based on the combination of ssGSEA and kTSP scores.

### Statistical analysis

Student *t*-tests were used for statistical analysis between two groups in in vitro experiments. For in vivo studies, natural logarithm (ln) transformation was performed on tumor volume and regression models were created for tumor volume by study day per animal. Analysis of variance was run on the slopes obtained from the regression analysis, and the Tukey–Cramer method was used to compare treatment groups. Statistical analyses were performed using GraphPad Prism software. Statistical analysis of the RNA-seq data was performed using DESeq2 package in R. DE genes were identified using the Wald test [45], with three mice per condition/treatment. Multiple hypothesis correction was performed using FDR [49], where significance was defined as FDR <0.05.

## Results

### AL101 inhibits cell viability in NOTCH1-mutated ACC organoid but not in NOTCH wild-type ACC cell lines

Due to the rarity of ACC, its slow growth rate, and the limited number of passages that primary ACC cells typically grow in vitro, ACC cell lines have rarely been established. Although approximately 10 ACC cell lines have been reported to date and some were experimentally used, the origin of most of these cell lines has been questioned [62–64]. The naturally immortalized HACC2A and UFH2 cell lines [65, 66], as well as hTERT-transformed ACC52 cells [67], are among the few available authenticated salivary ACC cell lines. As whole-exome sequencing (WES) of these cell lines did not reveal any activating mutations in *NOTCH* genes (not shown), we established an organoid model (Supplementary Fig. 1A) using surgical resection material from a patient with an ACC tumor carrying two activating mutations in *NOTCH1*, as detected by the OncoPlus cancer mutation panel [68]. Specifically, a missense mutation (c.4787T>C, p.L1586P) that disrupts the structure of the NRR, leading to ligand-independent production of NICD1; and a complex change in exon 34 of *NOTCH1* (c.7283_7286delinsCAG) that produces a coding frameshift starting at amino acid 2428 and creates a premature stop codon after 7 new amino acids, thereby leading to loss of a C-terminal PEST degron domain and stabilization of NICD1 [69]. Organoid histology and NICD1 expression were similar to those seen in the parental tumor (Supplementary Fig. 1B) and persistence of both *NOTCH1* mutations in the organoid model was confirmed by WES.

**Fig. 1:**
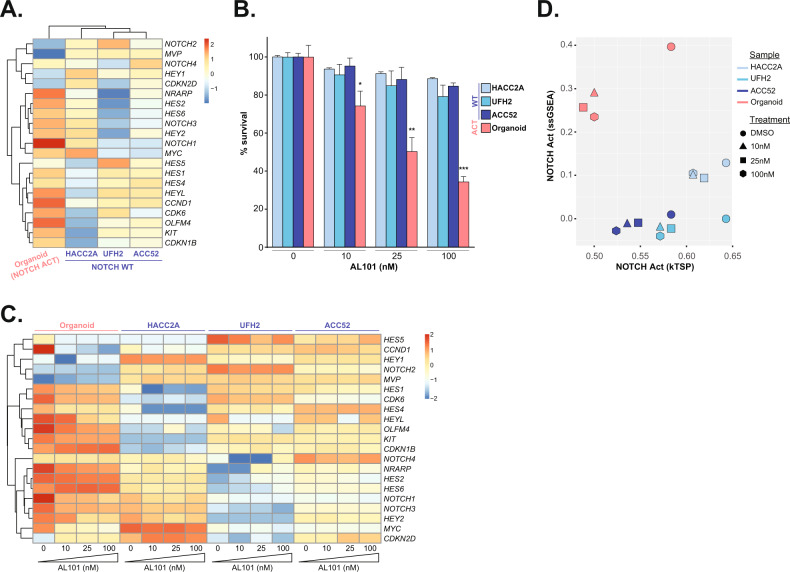
AL101 inhibits NOTCH activation and cell viability in a NOTCH1-mutated ACC organoid but not in NOTCH wild-type ACC cell lines. **A** Hierarchical clustering of normalized baseline expression of 21 NOTCH-related genes in an organoid bearing *NOTCH1* activating mutations and three ACC cell lines with wild-type *NOTCH* genes displayed as a heatmap. Higher or lower expression of genes is indicated with shades of red or blue cells, respectively. **B** In vitro ACC cell models were treated with increasing concentrations of AL101 and relative cell viability was determined on day 14. *p < 0.05, **p < 0.01, and ***p < 0.001. **C** Normalized expression of 21 NOTCH-related genes in the ACC in vitro cell models treated with increasing concentrations of AL101 displayed as a heatmap. Higher or lower expression of genes is indicated with shades of red or blue cells, respectively. **D** NOTCH activation caller plot scores the level of NOTCH signaling activity in the indicated samples by using ssGSEA (axis Y) and kTSP (axis X) algorithms on a set of NOTCH-related genes, where a shift down and to the left indicates reduced NOTCH signaling activity.

RNA-seq revealed that expression of most of the 21 known NOTCH-related genes was substantially higher in the organoid model harboring activating *NOTCH1* mutation compared to the three ACC cell lines bearing WT NOTCH genes (Fig. 1A). Reflecting the NOTCH signaling status, AL101 significantly inhibited the survival of the organoid model in a dose-dependent manner (Supplementary Fig. 2A), whereas only a minor response to AL101 was seen in the three WT ACC cell lines (Fig. 1B). Furthermore, treatment of the organoid model with AL101 resulted in downregulation of NICD1 (Supplementary Fig. 2B) and multiple NOTCH downstream target genes, such as *HES1*, *HES2*, *HEY2*, *HEYL*, and *NRARP*; NOTCH-dependent regulators of cell-cycle progression such as *CDK6* and *CCND1*; and pro-survival oncogenes such as *KIT* and *MYC* (Fig. 1C), confirming that NOTCH signaling was functionally inhibited. In parallel, tumor suppressor genes such as *MVP* and cyclin-dependent kinase inhibitors *CDKN1B* and *CDKN2D* were upregulated following AL101 treatment (Fig. 1C). When using the NOTCH caller to visualize expression levels of the *NOTCH* target genes, a striking reduction in NOTCH signaling was observed in the organoid model harboring *NOTCH1* activating mutations and not in *NOTCH1* WT ACC cell lines (Fig. 1D).

### Characterization of ACC patient-derived xenograft (PDX) models

**Fig. 2:**
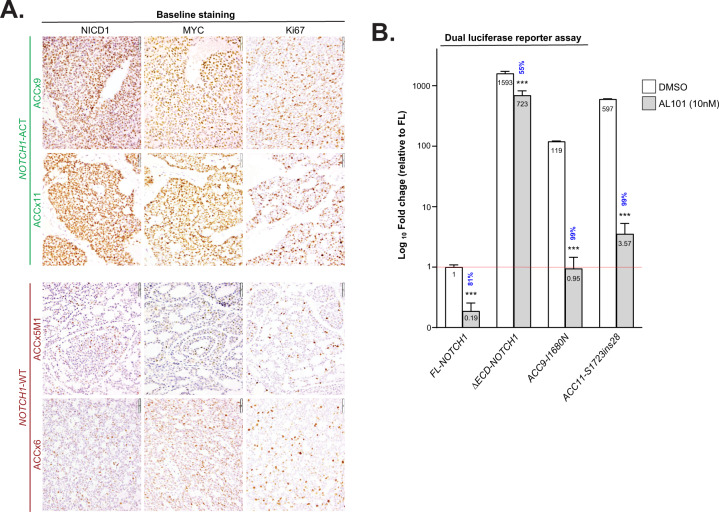
*NOTCH1* mutations in ACCx9 and ACCx11 PDX tumors show high levels of NOTCH1 signaling that is inhibited by AL101. **A** Tumor sections from four PDX models (ACCx9, ACCx11, ACCx5M1, and ACC6) collected at baseline (before implantation) were stained for NICD1, MYC, and Ki67 using an IHC. Representative images are shown. **B** Transactivation activity of wild-type full-length *NOTCH1* (*FL-NOTCH1*), constitutively active γ-secretase dependent form of the *NOTCH1* receptor (*ΔECD-NOTCH1*), and alleles containing mutations carried by ACCx9 and ACCx11 PDX tumors (*ACC9-I1680N* and *ACC11-S1723ins28*, respectively) was assessed using a dual-luciferase reporter assay. Bars represent the average of five independent experiments performed in triplicates and indicate fold-change relative to *FL-NOTCH1* (red line). Percent inhibition induced by AL101 (gray bars) relative to DMSO (white bars) is indicated in blue. ***p < 0.001.

To evaluate the effect of AL101 in vivo, we selected two ACC PDX models reported to harbor *NOTCH1* activating alterations: ACCx9, which carries an activating I1680N mutation; and ACCx11, which harbors a tandem duplication 3′ of *NOTCH1* and has high levels of NICD1 [15]. We also selected two models without activating aberrations in NOTCH genes, ACCx6, and ACCx5M1. The NOTCH1 signaling activation status of these four ACC tumor models was confirmed by IHC analysis (Fig. 2A). As expected, strong diffuse nuclear staining for NICD1, a hallmark of NOTCH1 signaling activation, was observed in ACCx9 and ACCx11 tumors, whereas staining for NICD1 in ACCx6 and ACCx5M1 models, which have biphasic growth patterns, was weaker and confined to a subset of cells. Expression of MYC, a well-known oncogene and NOTCH target gene [70], and Ki67 (a marker of cell proliferation) were also significantly elevated in ACCx9 and ACCx11 PDX models (Fig. 2A).

### Detection of NOTCH1 intronic retention in ACCx11

The tandem repeat that lies 3’ of *NOTCH1* in ACCx11 does not disrupt the NRR or PEST coding region, raising the possibility that some other genomic abnormality might explain the high levels of NICD1 production in this model. Since sequencing of exonic DNA failed to detect activating mutations in *NOTCH1*, we turned to RNA-seq, which revealed a tandem duplication involving a sequence normally encoded by exon 27. This tandem duplication results in the insertion of 28 amino acids between the structured part of the NRR and the transmembrane domain, creating a new “unprotected” ADAM10-cleavage site (Supplementary Fig. 3). Similar in-frame insertions of 11–36 amino acids stemming from internal tandem duplications in the 3’ end of intron 27 and/or the proximal region of exon 28 (*NOTCH1* extracellular juxtamembrane region), called juxtamembrane expansion (JME) mutations, have been reported in T-ALL and ACC and are known to result in a strong constitutive production of NICD1 [71, 72]. Since the NOTCH1 exon 27 was wildtype, we reasoned that the additional sequence must be encoded by an aberrant “new” exon in intron 27. Indeed, DNA sequencing of intron 27 revealed an insertion, dubbed exon 27a, flanked by splice donor and acceptor sites encoding the duplicated sequence (Supplementary Fig. 3). This type of mutation, which to our knowledge has not been reported previously, is consistent with the idea that tumors with high constitutive levels of NICD1 harbor mutations that disrupt the NOTCH NRR and/or PEST domain.

### NOTCH1 mutations in ACCx9 and ACCx11 tumors are activating and sensitive to AL101

To confirm the functional relevance of *NOTCH1* variants expressed by ACCx9 and ACCx11 tumors and their sensitivity to AL101 inhibition, U2OS cells were transfected with a NOTCH reporter gene and cDNAs encoding full-length WT *NOTCH1* (*FL-NOTCH1*), a strong gain-of-function form of *NOTCH1* with a truncated extracellular domain *(ΔECD-NOTCH1*), the ACCx9 NRR variant (*ACCx9-I1680N*), or the ACCx11 NRR variant (*ACCx11-S1723ins27a*), in the presence and absence of 10 nM AL101. Importantly, like *ΔECD-NOTCH1*, the *ACCx9-I1680N* and *ACCx11-S1723ins27a* mutant alleles produced >100-fold higher activation of the NOTCH1 reporter gene than the *FL-NOTCH1* allele (Fig. 2B). Furthermore, AL101 resulted in a marked reduction of NOTCH1 signaling in cells expressing the *ACCx9-I1680N* and *ACCx11-S1723ins27a* variants (99.2 and 99.4% inhibition, respectively) (Fig. 2B), confirming the dependency of these polypeptides on γ-secretase cleavage for NICD1 production and validating the use of these models in subsequent in vivo studies.

### Tolerability of AL101 in vivo

Intravenous and oral administration of AL101 at a minimum effective dose (1 mg/kg) [22] had very similar PK profiles in mice (Supplementary Fig. 4A), and we therefore used oral administration in all in vivo experiments. To confirm AL101 tolerability in tumor-free mice, animals were treated with 3 escalating doses of AL101 (3, 5, or 7.5 mg/kg; 4 days on/3 days off) for 2 weeks. We observed that AL101 was well tolerated, with no overt toxicity (as assessed by altered behavior/appearance, or significant weight loss) (Supplementary Fig. 4B), consistent with prior reports showing that AL101 is well tolerated in vivo [22].

### AL101 inhibits tumor growth in NOTCH1-activated ACC PDX models

**Fig. 3:**
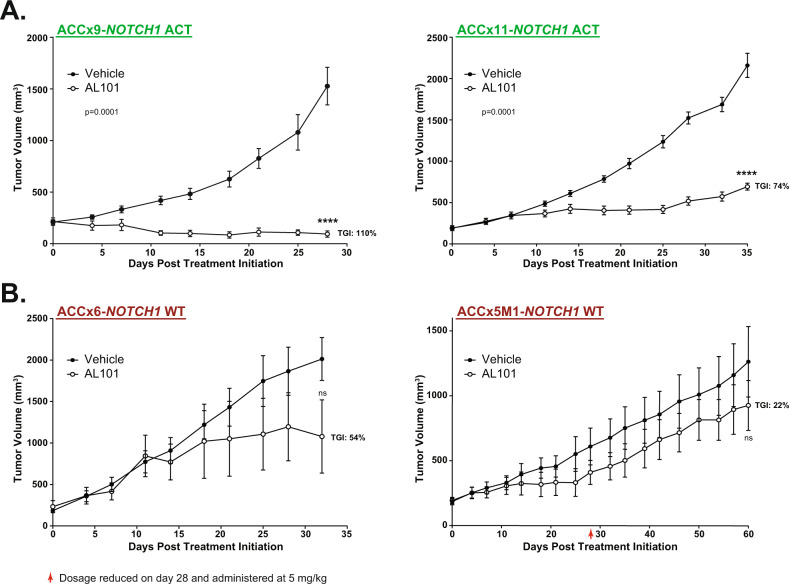
AL101 induces tumor growth inhibition in NOTCH1-mutated ACC PDX models. Two ACC PDX models harboring tumors with *NOTCH1* activating alterations (NOTCH-Act) (**A**) and two PDX models without NOTCH-activating mutations (NOTCH WT) (**B**) were treated with either AL101 or vehicle. Graphs show the average tumor volume for five to ten animals ±SD. Red arrow: dosage reduced on day 28 and administered at 5 mg/kg. **p < 0.01, ****p < 0.001. Act activated *NOTCH1*, WT wild-type *NOTCH1*.

To determine the impact of AL101 on tumor growth in vivo, animals were subcutaneously implanted with each of the 4 ACC tumor models and treated with either 7.5 mg/kg of AL101 or vehicle on a 4 day on/3 day off schedule. Tumor volumes and body weight were assessed twice a week, and percentage TGI was calculated as described in the Methods section. Although a long-term 7.5 mg/kg dosing schedule was tolerated in the tumor-bearing animals, with an observed weight loss of <10% (Supplementary Fig. 5), a slight decrease in body weight occurred at three consecutive timepoints (days 22, 25, and 28) in ACCx5M1 model (Supplementary Fig. 5). Thus, the dosage in this model was reduced to 5 mg/kg and maintained at this level until the end of the experiment. Importantly, while AL101 therapy induced potent TGI in both models with *NOTCH1* gain-of-function mutations (110 and 74% for ACCx9 and ACCx11, respectively; *p* < 0.0001) (Fig. 3A and Supplementary Fig. 6), no significant antitumor effect was seen in models lacking NOTCH-activating aberrations (Fig. 3B and Supplementary Fig. 6). Notably, treatment of animals with 3 mg/kg of AL101, the lowest dose evaluated for tolerability in vivo (Supplementary Fig. 4), produced TGIs (Supplementary Fig. 7) and had effects on body weights (Supplementary Fig. 8) that were similar to those induced by treatment with a 7.5 mg/kg dose, suggesting that AL101 has a fairly broad therapeutic window for treating ACC cancers driven by activated *NOTCH1*.

### AL101-induced tumor growth inhibition is associated with a decrease in NOTCH-mediated tumorigenic signaling

**Fig. 4:**
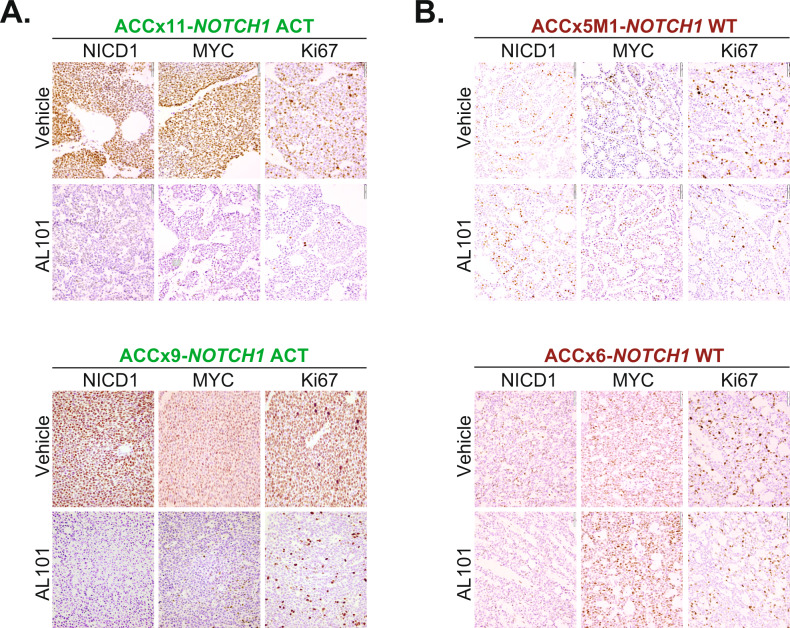
AL101-induced tumor growth inhibition in vivo is associated with a decrease in NICD1, MYC, and Ki67 expression. Tumor sections derived from ACCx9 and ACCx11 (**A**) or ACCx5M1 and ACCx9 **B** xenograft model treated with either AL101 or vehicle were stained for NICD1, MYC, and Ki67 using an IHC. Representative images are shown. Act activated *NOTCH1*, WT wild-type *NOTCH1*.

**Fig. 5:**
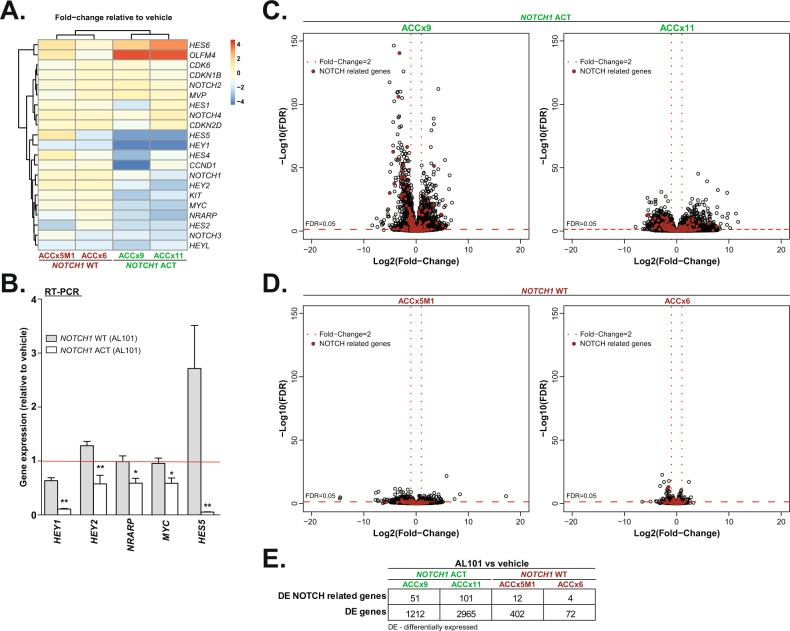
AL101 inhibits NOTCH-mediated tumorigenic signaling in vivo. **A** Fold-change in expression of 21 NOTCH-related genes (from normalized RNA-seq data) in animals treated with AL101 relative to vehicle-treated animals, displayed as a hierarchical clustering heatmap. Increased and reduced expression of genes is indicated with shades of red or blue cells, respectively. **B** Confirmation of RNA-seq data with RT-PCR analysis of selected NOTCH target genes in AL101-treated relative to vehicle-treated animals (red line). Gray bars: animals carrying wild-type NOTCH1 alleles; white bars: animals harboring *NOTCH1* activating mutations. *p < 0.05, and ***p < 0.01. **C**, **D** Volcano plots depicting gene expression changes in AL101 versus vehicle-treated animals. The red circles represent genes included in a 478 NOTCH-related geneset curated from KEGG, PID, MSigDB, and PathwayCommons databases (see Supplementary Table 1 for details). **E** The table summarizes the total number of differentially expressed (DE) genes and NOTCH-related DE genes in AL101-treated animals relative to vehicle-treated counterparts.

Consistent with TGI, NICD1, MYC, and Ki67 protein levels were sharply reduced by AL101 treatment in tumors with constitutively active *NOTCH1* alleles (ACCx9 and ACCx11), but not in tumors with WT *NOTCH1* (ACCx5M1 and ACCx6) (Fig. 4). Moreover, RNA-seq revealed that expression of 21 NOTCH-related genes was substantially altered by AL101 in *NOTCH1*-mutated models as compared to NOTCH WT counterparts (Fig. 5A). These results were further validated by RT-PCR analysis of five selected NOTCH-related genes (Fig. 5B). As NOTCH signaling involves a highly interconnected and complex network of protein modifiers that can affect its activity [73], we generated a set of 478 NOTCH-related genes (taken from KEGG, PID, MSigDB, and PathwayCommons databases) that may be affected by γ-secretase inhibition (Supplementary Table 1). Notably, in response to AL101 treatment, a substantially higher number of NOTCH-related genes, as well as a total number of genes, were DE in *NOTCH1*-mutated tumors compared to NOTCH WT specimens (Fig. 5E). Furthermore, downregulated genes (colored blue in Supplementary Fig. 9A, B) in AL101-treated ACCx11 and ACCx9 tumors were significantly enriched (*p* value < 10^−16^, exact binomial test) in “pathways in cancer”, a KEGG pathway (hsa05200) that contains 325 known tumor driving genes, suggesting that inhibition of NOTCH signaling has broad anti-oncogenic effects in ACC.

## Discussion

Despite a relatively indolent growth rate, late relapse and distant metastasis occur in approximately 50% of patients with ACC [1]. To date, there are no FDA-approved targeted agents for patients with ACC, and responses to conventional chemotherapeutic agents are limited.

Cumulative evidence suggests that ~20% of ACC tumors carry mutations in *NOTCH1*, a critical regulator of cell proliferation, differentiation, and survival [7, 8, 14, 15], and that ACCs with alterations involving portions of *NOTCH1* encoding the NRR and PEST domains are associated with worse prognosis [7, 8, 15, 72, 74, 75]. Our studies here indicate that ACCs with aberrant NOTCH signaling driven by activating genetic alterations are sensitive to AL101, a GSI that potently and specifically inhibits all 4 NOTCH receptors at nanomolar concentrations [22, 37, 76, 77], providing strong preclinical evidence for the ongoing Phase II open-label, single-arm, multicenter study (ACCURACY; NCT03691207) in relapsed/refractory ACC patients harboring activating NOTCH alterations.

A major challenge in the development of new therapeutic approaches for ACC has been the limited number of appropriate cellular and animal models. Developing ACC cell lines has been challenging, and to date, there are only five cell lines authenticated as ACC [63, 64, 66, 78, 79]. None of the three ACC cell lines that were available for this study (HACC2A, UFH2, and ACC52) harbored a NOTCH-activating mutation and consequently have low endogenous NOTCH signaling activity. Identification of the population of cancer stem cells in ACC and their ability to form cellular aggregates [74, 80, 81] makes this tumor an excellent candidate for establishing three-dimensional organoid models. While organoids remain genetically and phenotypically stable and maintain predictive value for drug responses of individual patients [82, 83], due to the rarity of the disease, the use of ACC organoids remains extremely limited [84], and no cellular model bearing a NOTCH-activating alteration is currently available. To study the effect of AL101 ex vivo, we established a human organoid model from a surgically excised ACC bearing a confirmed *NOTCH1* activating mutation. We observed that AL101 had potent dose-dependent inhibitory effects on growth and NOTCH target gene expression in this model, whereas limited effects were seen in three ACC cell lines with WT *NOTCH1*. This suggests that the antitumor activity of AL101 may be restricted to ACC tumors with a constitutively activated NOTCH pathway.

To further test this idea, we studied the activity of AL101 in ACC PDX models. It is established that PDX models typically maintain the histology, gene expression patterns, and drug response of the tumors from which they were derived [85]. We selected 4 PDX models of known *NOTCH1* mutation/activation status that were previously used to assess the efficacy of brontictuzumab, a monoclonal antibody that inhibits NOTCH1 by binding to its extracellular domain [15]. Although brontictuzumab had an inhibitory effect against ACCx9 and ACCx11, it was less impressive than the activity of AL101, possibly because mutations that altered the structure of the *NOTCH1* NRR, particularly the type of JME mutation found in ACCx11, may abrogate the ability of blocking antibodies to prevent ADAM10 cleavage [86], the first step in NICD1 generation. This limitation argues that GSIs may be more generally reliable in targeting mutated NOTCH receptors than blocking antibodies. A second practical concern raised by the unusual mutation found in ACCx11 is that such mutations will be missed with standard exome sequencing, suggesting that RNA-seq platforms such as the Tempus xT genomic profiling test (https://www.ncbi.nlm.nih.gov/gtr/tests/558436) may have greater sensitivity in identifying *NOTCH* gain of function mutations. Our experience with ACCx11 also highlights the utility of immunohistochemical analysis for NICD1, as diffuse NICD1 staining in ACC is strongly correlated with the presence of *NOTCH1* activating aberrations [72].

Treatment of tumor-bearing mice with AL101 led to significant responses in PDX models with activating *NOTCH1* mutations but not in models with WT *NOTCH* genes. Responses were paralleled by a reduction in activated NOTCH1 and expression of NOTCH downstream target genes, as demonstrated by IHC, RNA-seq and RT-PCR analyses, with no significant toxicity observed in AL101-treated mice. These results further support the use of biomarkers that correlate with constitutive NOTCH activation to identify tumors that are likely to respond to AL101 and other NOTCH inhibitors [15, 50, 87]. While further studies in additional ACC in vitro models (once established) and PDXs are required to determine whether mutation-independent NOTCH pathway activation may also help predict response to NOTCH inhibitors, particularly when used in combination with other agents [37, 63, 77], our data emphasize the need to carefully assess ACCs for *NOTCH1* activating mutations when selecting patients for treatment with GSIs.

In conclusion, our work adds to the growing body of evidence underscoring the critical role of NOTCH pathway activation in aggressive subtypes of ACC [15] and provides further support for the clinical development of GSIs for this indication. An ongoing Phase II ACCURACY clinical trial [88], which is assessing the effect of AL101 in patients with ACCs bearing NOTCH-activating mutations, is showing promising clinical activity and a favorable safety profile. Future extensions of the studies described here and ongoing clinical trials are likely to revolve around the identification of rational combinations of GSIs and other agents that increase tumor response and diminish the emergence of resistance to GSIs [89].

## Supplementary information

Supplementary Figure 1

Supplementary Figure 2

Supplementary Figure 3

Supplementary Figure 4

Supplementary Figure 5

Supplementary Figure 6

Supplementary Figure 7

Supplementary Figure 8

Supplementary Figure 9

Supplementary Table 1

Checklist

## Data Availability

The data supporting the findings of this study are available within the article and its Supplementary information files.
