# Supplementary material for: AL101, a gamma-secretase inhibitor, has potent antitumor activity against adenoid cystic carcinoma with activated NOTCH signaling
Source: Cell Death Dis. 2022 Aug 5;13(8):678. doi: 10.1038/s41419-022-05133-9 (PMC9355983; doi:10.1038/s41419-022-05133-9)
Supplement: Supplementary file 1 — Supplementary Figure 1 [file 41419_2022_5133_MOESM1_ESM.pdf]

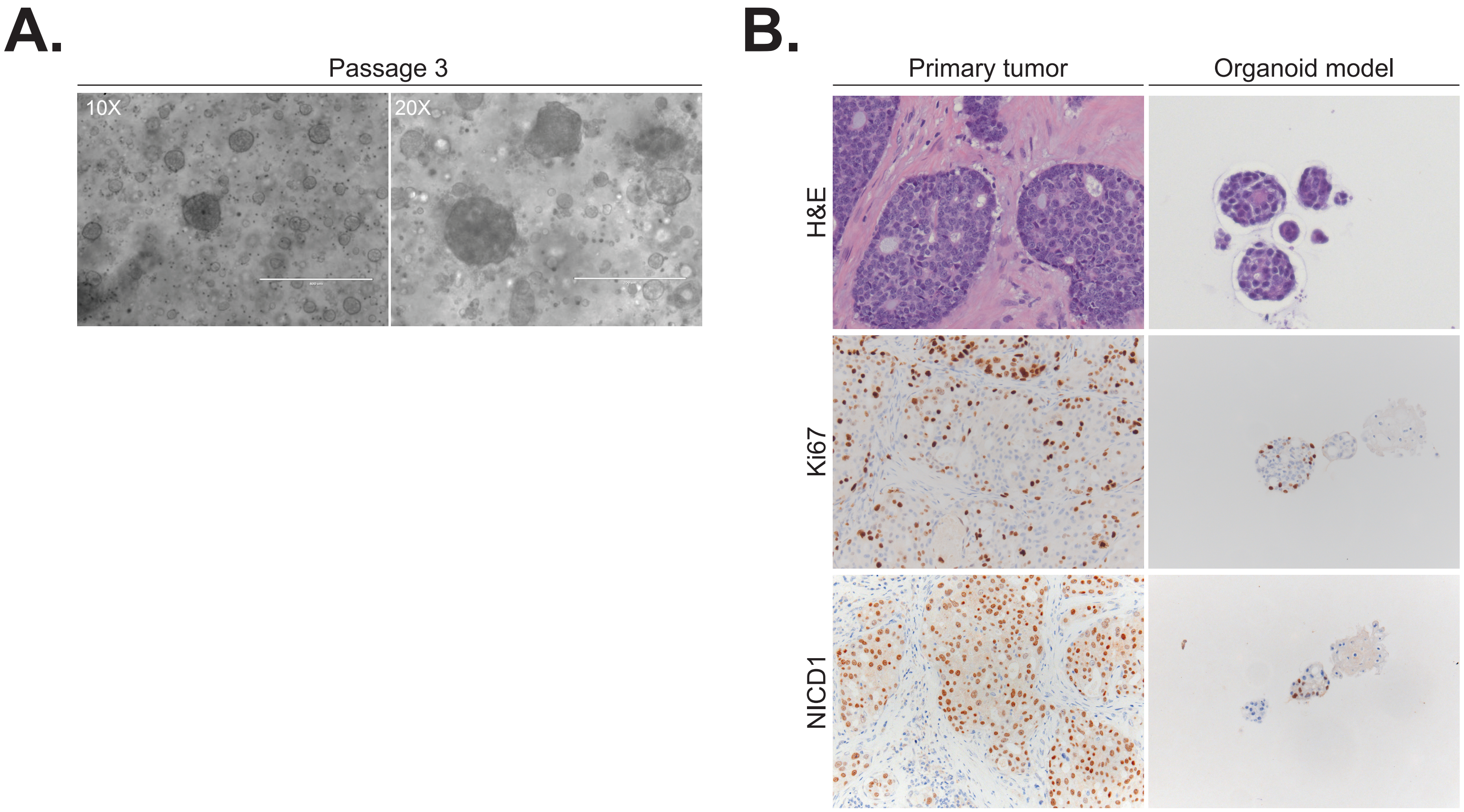

Supplementary Figure 1. A. Phase-contrast images showing organoid growth at passage 3, which was used for experiments reported in this study. **B.** Hematoxylin and eosin (H&E), Ki67 and NICD1 staining of an organoid model at passage 3 and a primary ACC tumor it was established from.
