# Supplementary material for: AL101, a gamma-secretase inhibitor, has potent antitumor activity against adenoid cystic carcinoma with activated NOTCH signaling
Source: Cell Death Dis. 2022 Aug 5;13(8):678. doi: 10.1038/s41419-022-05133-9 (PMC9355983; doi:10.1038/s41419-022-05133-9)
Supplement: Supplementary file 2 — Supplementary Figure 2 [file 41419_2022_5133_MOESM2_ESM.pdf]

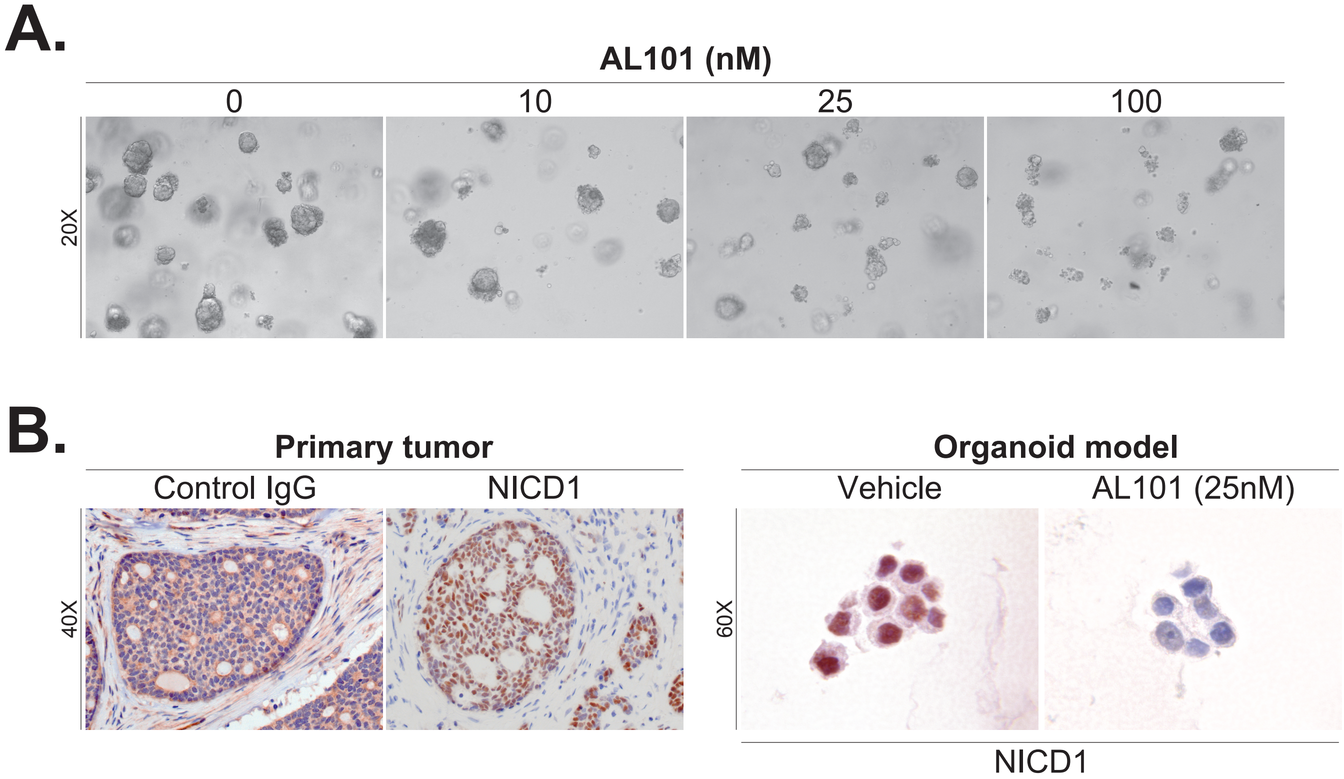

Supplementary Figure 2. A. Representative brightfield images of *NOTCH1* mutant organoid model treated with increasing doses of AL101 for 10 days. **B.** Left - staining of the primary ACC tumor with either non-specific IgG antibody (control) or anti-NICD1 antibody. Right - NICD1 staining of the *NOTCH1* mutant organoid model treated with either vehicle (control) or 25nM AL101 for 10 days.
