# Supplementary material for: AL101, a gamma-secretase inhibitor, has potent antitumor activity against adenoid cystic carcinoma with activated NOTCH signaling
Source: Cell Death Dis. 2022 Aug 5;13(8):678. doi: 10.1038/s41419-022-05133-9 (PMC9355983; doi:10.1038/s41419-022-05133-9)
Supplement: Supplementary file 3 — Supplementary Figure 3 [file 41419_2022_5133_MOESM3_ESM.pdf]

Transcript sequence

CTCCATCGTCTACCTGGAGATTGACAACCGGCAGTGTGTGCAGGCCTCCTCGCAGTGCTTCCAG
AGTGCCACCGATGTGGCCGCATTCCTGGGAGCGCTCGCCTCGCTGGGCAGCCTCAACATCCCC
TACAAGATCGAGGCCGTGCAGAGCGGTCACAGCTGCCGGCCACCTGGGAACGAGGCGGTCACA
GCTGCCGGTGGAGCCACCTGGGAACGAGTCCAGCCACGGGAAAGTGAGACCGTGGAGCCGCC
CCCGCCGGCGCAGCTGCACTTCATGTACGTGGCGGCGGCCGCCTTTGTGCTTCTGTTCTTCGTG
GGCTGCGGGGTGCTGCTGTCCCGCAAGCGCCGGCGGCAGCATGGCCAGCTCTGGTTCCCTGA
GGGCTTC

Green – exon 27 (E27)
Brown – insertion
Blue – exon 28 (E28)

Genomic sequence

CTCCATCGTCTACCTGGAGATTGACAACCGGCAGTGTGTGCAGGCCTCCTCGCAGTGCTTCCAG
AGTGCCACCGACGTGGCCGCATTCCTGGGAGCGCTCGCCTCGCTGGGCAGCCTCAACATCCCC
TACAAGATCGAGGCCGTGCAGA**GT**AAGTGTGGCCCCATCCCGGGAACAGGCTCTGCCTGCAGG
I27a GGGTGCCATCCCCCCGTGCCCCAGACACGCTGGCTGTTTGTGCCAGTTGCTACCCACGGGTGT
GAGCGTTGCCGTCCGAGTTGGGGTAGGGCTTTTCTGGAATTTTCTGAATGGCACTCCGCCCCCA
CCTGC**AG**GCGGTCACAGCTGCCGGCCACCTGGGAACGAGGCGGTCACAGCTGCCGGTGGAGC
CACCTGGGAACGAGTCCAGCCACGGGAAA**GT**GGGTGCCTGCTTCTCTCCCCACCCTTTCCTCCT
GAATTTTCTTTGTTGGGTATTATTTCAAAATCATTACGGCTTTTTTTAAAGAAAAAAAAAGAGAGAG
AGAGAAGAATTGATCGGTGTCATGTGAAGTGTTGAAGTTTGTATCTTGAAAATCCCTCTAAATCCT
TTGTCTTAACAGCTCAGTGCGAGTGCAGCGATTTGAAGTTGACTAATCCTCCTTCCTTAAAGGAG
I28a AAAAAAGTAAAAGCCGTCTCCAGATAGAGTCGGCTGGTGCAGGAGAGAATTTAGCGATAGTTTG
CAATTCTGATTAATCGCGTAGAAAATGACCTTATTTTGGAGGGCGGGATGGAGGAAATGGGGTTT
AAGAAGGCCCCCGGACGCGAAGCCAGTCCGCCGCCCCCCGGCCACCAGCCTGCTGCGTAGCC
GCTGCCTGATGTCCGGGCACCTGCCCCTGGCCCCCGTGCCCGC**AG**GTGAGACCGTGGAGCCG
CCCCCGCCGGCGCAGCTGCACTTCATGTACGTGGCGGCGGCCGCCTTTGTGCTTCTGTTCTTC
GTGGGCTGCGGGGTGCTGCTGTCCCGCAAGCGCCGGCGGCAGCATGGCCAGCTCTGGTTCCC
TGAGGGCTTCAAAGTGTCTGAGGCCAGCAAGAAGAAGCGGCGGGAGCCCCTCGGCGAGGACTC
CGTGGGCCTCAA

Green – exon 27 (E27)
Brown – exon 27a
Blue – exon 28 (E28)
I27a – intron 27a
I28a – intron 28a
Bold - splice donor and acceptor sites

Protein sequence encoded by the variant splice form

SIVYLEIDNRQCVQASSQCFQSATDVAAFLGALASLGSLNIPYKIEAVQSGHSCRPPGNEAVTAAGGA
TWERVQPRESETVEPPPPAQLHFMYVAAVEPPPPAQLHFMYVAA

Brown – inserted region

Supplementary Figure 3. Transcript, genomic and protein sequence of the NOTCH1 variant splice form identified in the ACCx11 PDX tumor.
