# Supplementary material for: AL101, a gamma-secretase inhibitor, has potent antitumor activity against adenoid cystic carcinoma with activated NOTCH signaling
Source: Cell Death Dis. 2022 Aug 5;13(8):678. doi: 10.1038/s41419-022-05133-9 (PMC9355983; doi:10.1038/s41419-022-05133-9)
Supplement: Supplementary file 4 — Supplementary Figure 4 [file 41419_2022_5133_MOESM4_ESM.pdf]

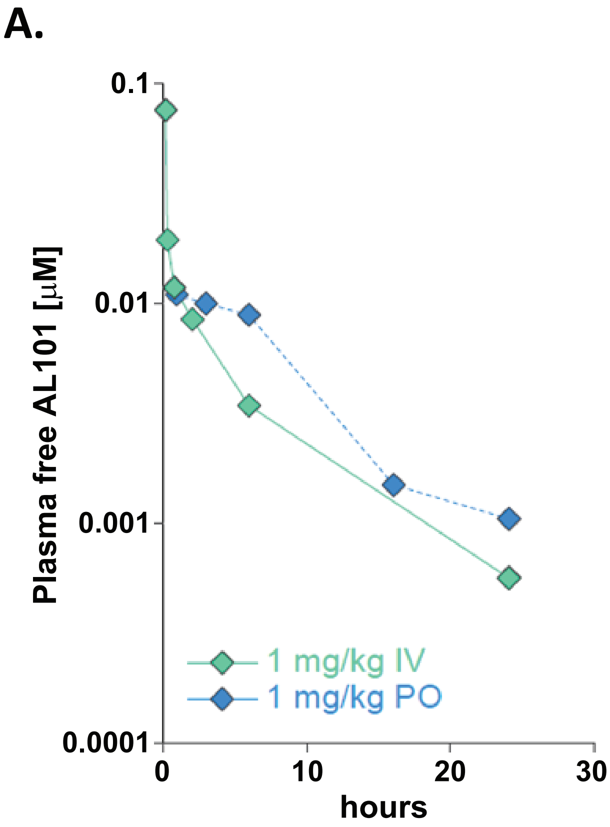

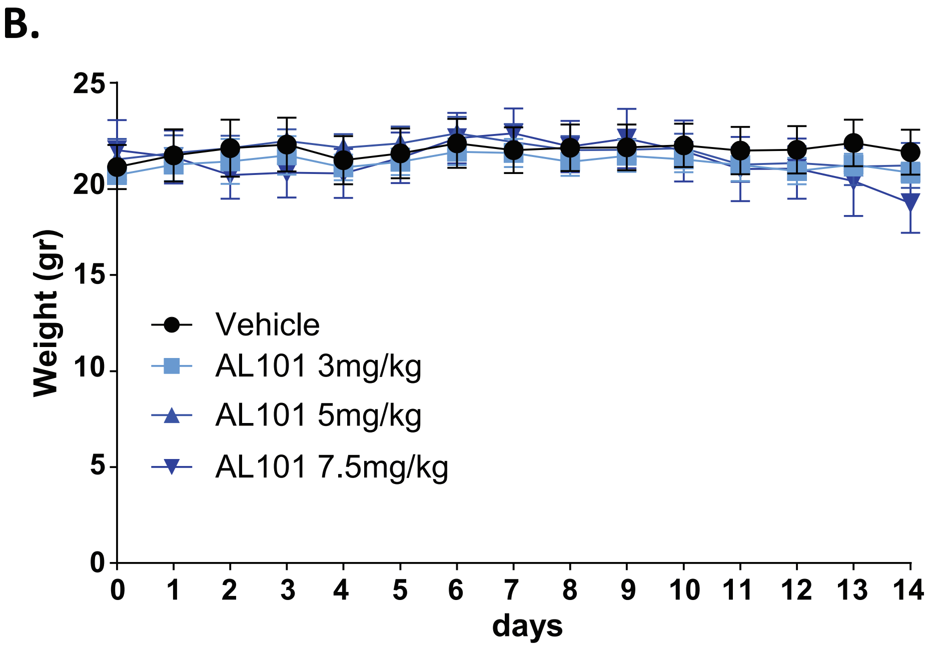

Supplementary Figure 4. A. Comparison of the pharmacokinetics of AL101 administered by two different routes [Intravenous (IV) and per os (PO)]. Plasma total free active drug concentrations in tumor-free mice was measured following the administration of AL101 at 1 mg/kg by either PO or IV. **B.** Escalating doses of AL101 were administered PO in tumor free animals for 2 weeks and body weight was assessed daily.
