# Supplementary material for: AL101, a gamma-secretase inhibitor, has potent antitumor activity against adenoid cystic carcinoma with activated NOTCH signaling
Source: Cell Death Dis. 2022 Aug 5;13(8):678. doi: 10.1038/s41419-022-05133-9 (PMC9355983; doi:10.1038/s41419-022-05133-9)
Supplement: Supplementary file 5 — Supplementary Figure 5 [file 41419_2022_5133_MOESM5_ESM.pdf]

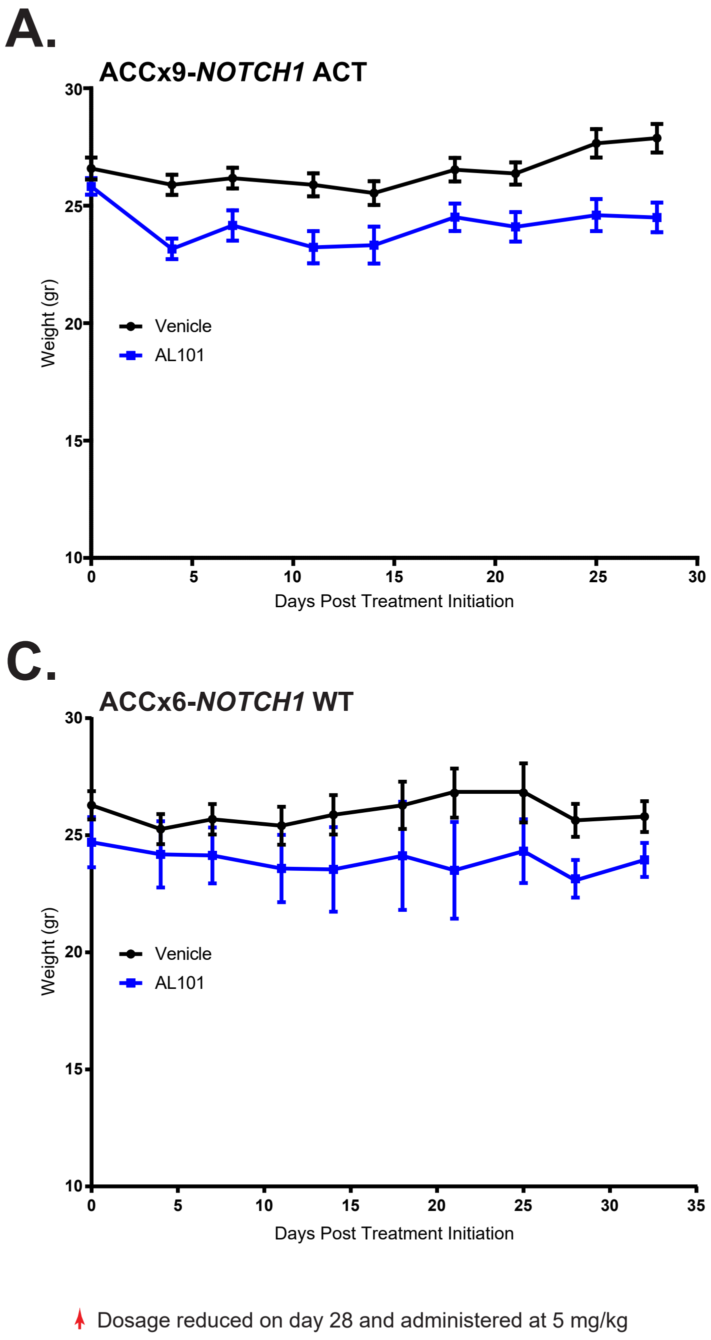

Supplementary Figure 5. Graphs show the average body weights for the PDX models used in this study that were treated with either AL101 (blue curves) or vehicle (black curves). Red arrow - dosage reduced on day 28 and administered at 5 mg/kg. ACT – activated *NOTCH1*. WT – wild type *NOTCH1*.
