# Supplementary material for: AL101, a gamma-secretase inhibitor, has potent antitumor activity against adenoid cystic carcinoma with activated NOTCH signaling
Source: Cell Death Dis. 2022 Aug 5;13(8):678. doi: 10.1038/s41419-022-05133-9 (PMC9355983; doi:10.1038/s41419-022-05133-9)
Supplement: Supplementary file 6 — Supplementary Figure 6 [file 41419_2022_5133_MOESM6_ESM.pdf]

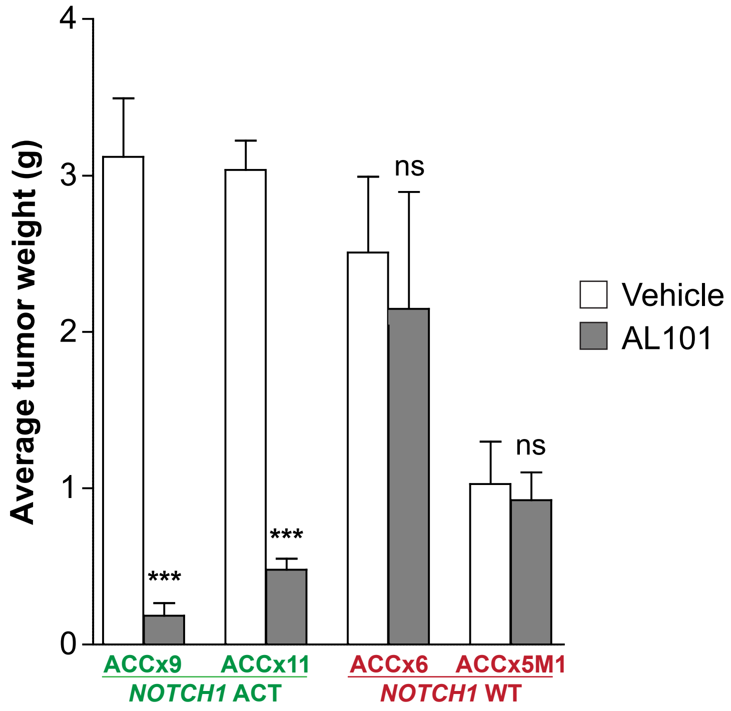

Supplementary Figure 6. Mean tumor weights at necropsy (± SD) in four PDX models treated with either vehicle or AL101. *** p<0.001, ns – not significant, ACT – activated *NOTCH1*, WT – wild type *NOTCH1*.
