# Supplementary material for: AL101, a gamma-secretase inhibitor, has potent antitumor activity against adenoid cystic carcinoma with activated NOTCH signaling
Source: Cell Death Dis. 2022 Aug 5;13(8):678. doi: 10.1038/s41419-022-05133-9 (PMC9355983; doi:10.1038/s41419-022-05133-9)
Supplement: Supplementary file 7 — Supplementary Figure 7 [file 41419_2022_5133_MOESM7_ESM.pdf]

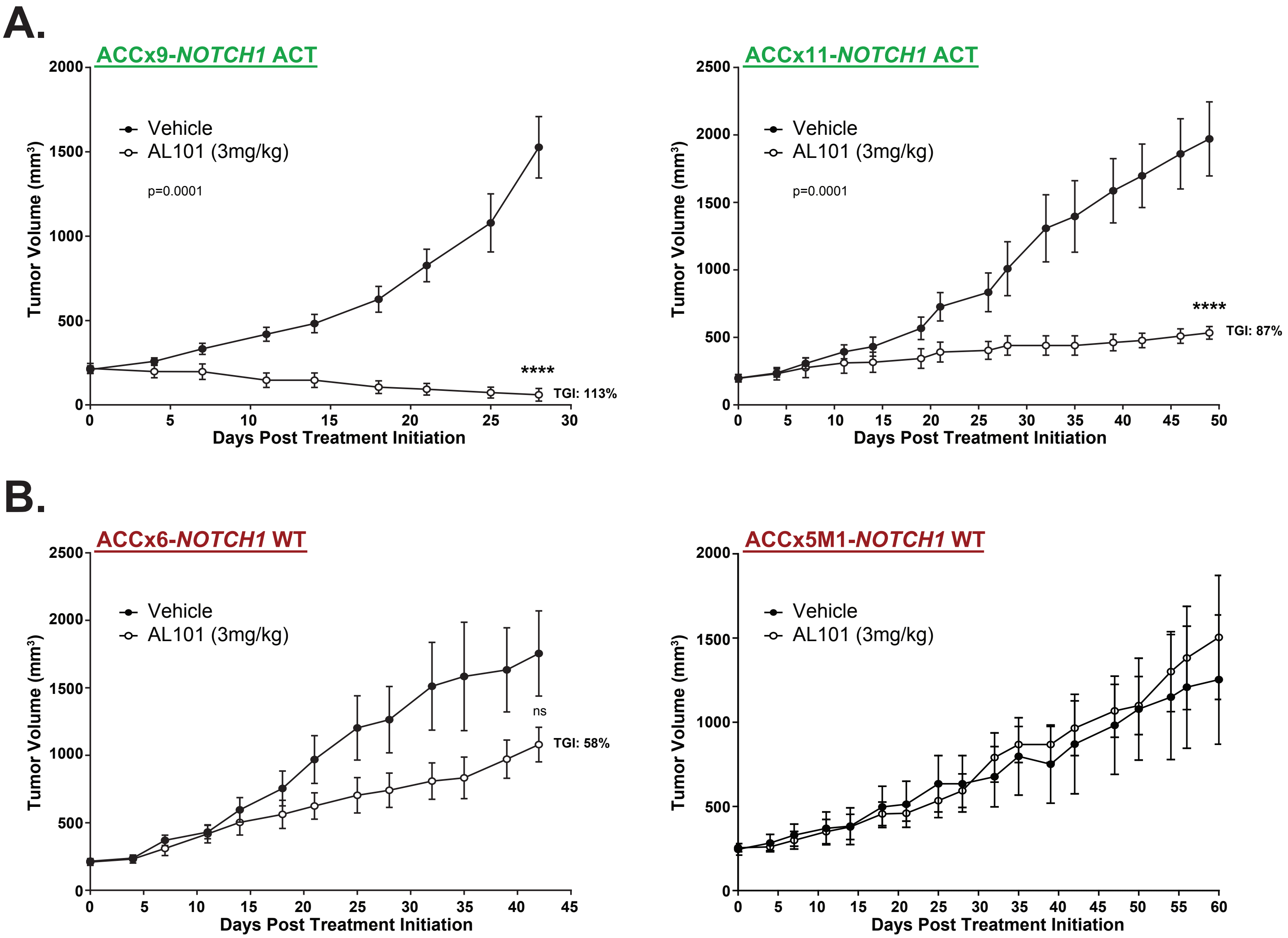


**Supplementary Figure 7.** Two ACC PDX models harboring tumors with *NOTCH1* activating alterations (*NOTCH1*-ACT) (**A**) and two PDX models without *NOTCH1* activating mutations (*NOTCH1*-WT) (**B**) were treated with either 3mg/kg AL101 or vehicle. Graphs show the average tumor volume for five to ten animals ± SD. **** p<0.001. ACT – activated *NOTCH1*. WT – wild type *NOTCH1*.
