# Supplementary material for: AL101, a gamma-secretase inhibitor, has potent antitumor activity against adenoid cystic carcinoma with activated NOTCH signaling
Source: Cell Death Dis. 2022 Aug 5;13(8):678. doi: 10.1038/s41419-022-05133-9 (PMC9355983; doi:10.1038/s41419-022-05133-9)
Supplement: Supplementary file 8 — Supplementary Figure 8 [file 41419_2022_5133_MOESM8_ESM.pdf]

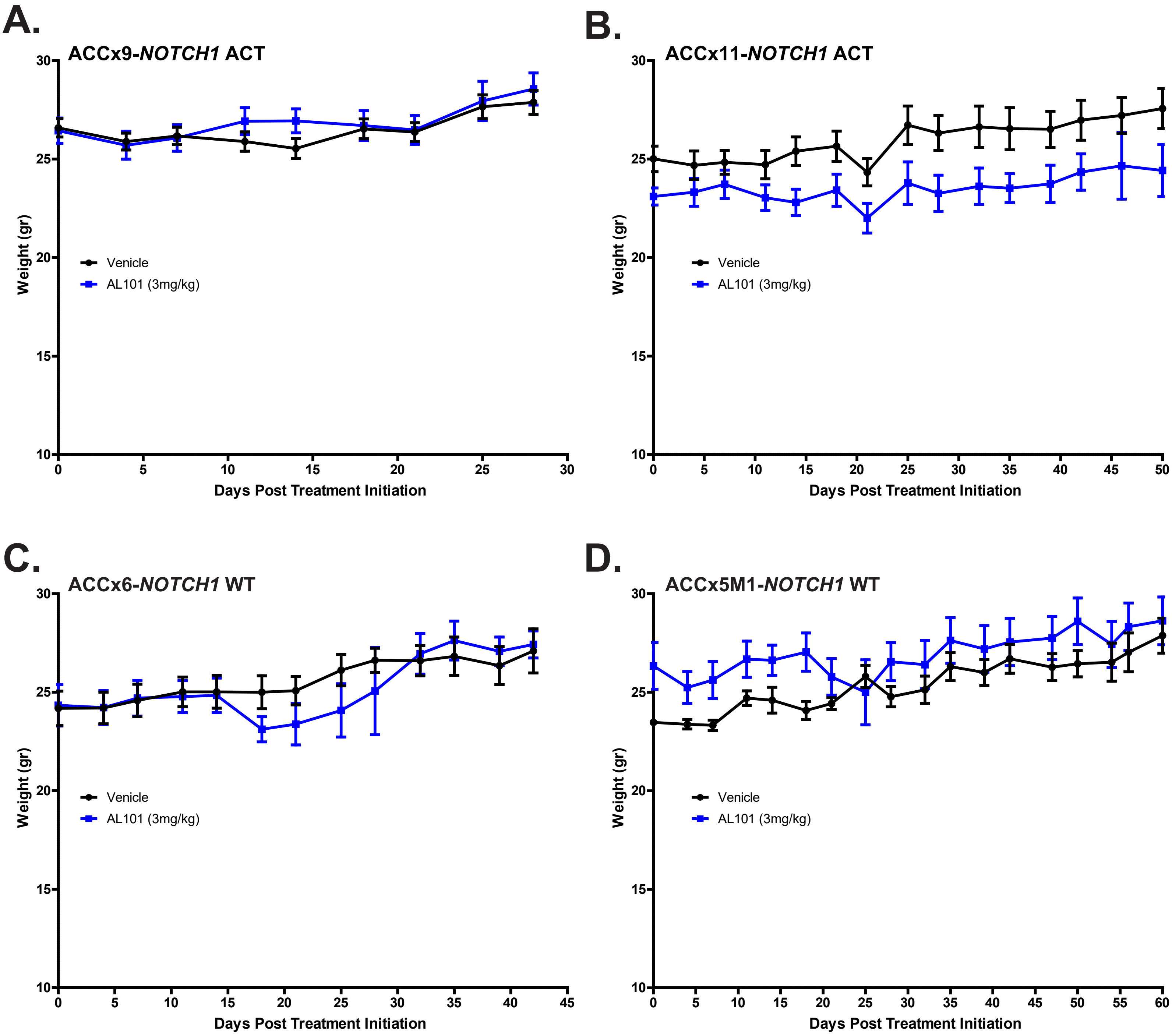

Supplementary Figure 8. Graphs show the average body weights for the PDX models treated with either AL101 (3 mg/kg) or vehicle (black curves). ACT – activated *NOTCH1*. WT – wild type *NOTCH1*.
