# Supplementary material for: AL101, a gamma-secretase inhibitor, has potent antitumor activity against adenoid cystic carcinoma with activated NOTCH signaling
Source: Cell Death Dis. 2022 Aug 5;13(8):678. doi: 10.1038/s41419-022-05133-9 (PMC9355983; doi:10.1038/s41419-022-05133-9)
Supplement: Supplementary file 9 — Supplementary Figure 9 [file 41419_2022_5133_MOESM9_ESM.pdf]

A. *NOTCH1* ACT — ACCx9, ACCx11

B. *NOTCH1* WT — ACCx5M1, ACCx6

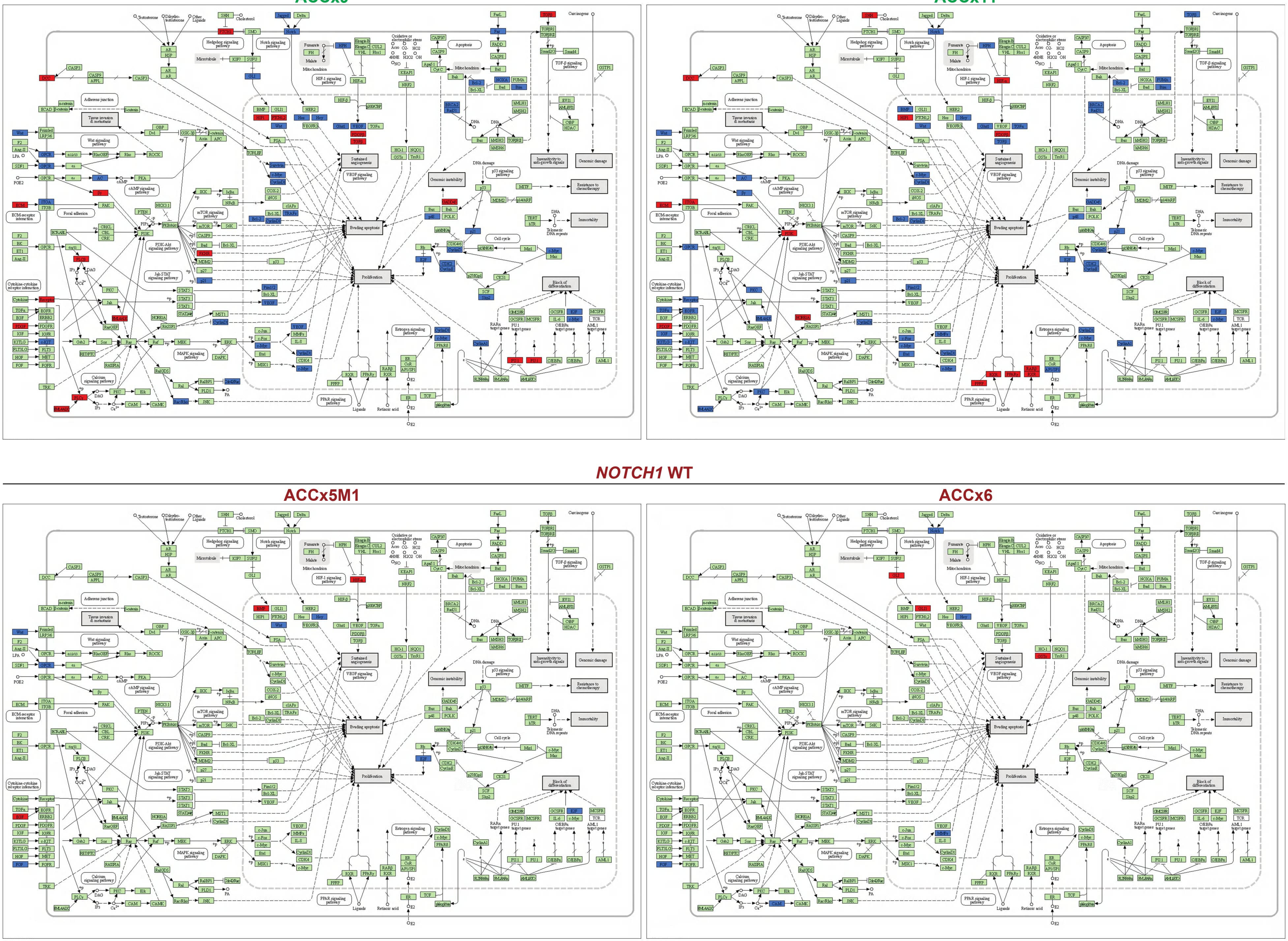

Supplementary Figure 9. Enrichment of AL101-induced differentially expressed genes in “pathways in cancer” (KEGG ID: hsa05200). Down-regulated genes are colored in blue. Up-regulated genes are colored in red. **A.** ACCx11 and ACCx9 tumors. **B.** ACCx5M1 and ACCx6 tumors.
