# Supplementary material for: AL101, a gamma-secretase inhibitor, has potent antitumor activity against adenoid cystic carcinoma with activated NOTCH signaling
Source: Cell Death Dis. 2022 Aug 5;13(8):678. doi: 10.1038/s41419-022-05133-9 (PMC9355983; doi:10.1038/s41419-022-05133-9)
Supplement: Supplementary file 10 — Supplementary Table 1 [file 41419_2022_5133_MOESM10_ESM.pdf]

DB	ID	Pathway/Gene-set name	Genes	# genes
PathwayCommons	--	NOTCH “controls-expression-of”	ACTA2, AKT1, APC, AXIN1, CCND1, CDH1, CDKN1A, CIR1, CTNNB1, DLGAP5, EGFR, FABP7, FCER2, FLT4, FOS, GATA3, GLI1, GZMB, H3F3A, H3F3B, HES1, HES5, HEY1, HEY2, HEYL, HIST1H3A, HIST1H3B, HIST1H3C, HIST1H3D, HIST1H3E, HIST1H3F, HIST1H3G, HIST1H3H, HIST1H3I, HIST1H3J, HIST2H3A, HIST2H3C, HIST2H3D, IL4, JUN, MMP2, MYC, NCOR2, NKX2-1, NOTCH1, NOTCH2, NOTCH3, NOTCH4, NTRK1, NUMB, NUMBL, PAQR4, PARP1, PAX8, PBRM1, PBX1, PCM1, PCMT1, PDIA6, PDS5A, PHF8, PIK3CA, PIK3CG, PIK3R1, PIK3R2, PIN1, PLAUR, PLXND1, POFUT1, POLR2A, POU2AF1, PPIA, PPP2CA, PPP2CB, PPP2R1A, PPP2R2A, PPP6R1, PPP6R3, PRKAA1, PRKCD, PRKDC, PRPF19, PRPF6, PRPF8, PRR20D, PSEN1, PSEN2, PSMA1, PSMB1, PSMC3IP, PTBP1, PTBP3, PTCRA, RAB5A, RAB7A, RABGGTB, RANBP10, RANBP9, RB1, RBBP4, RBM10, RBP3, RBPJ, RBPJL, RBX1, RDX, REL, RELA, RET, RFC1, RFC4, RFX1, RHOA, RIF1, RING1, RMC1, RMI2, RND3, RNF2, RNF4, RNF40, ROCK1, RPS27A, RPS6KB1, RTRAF, RUNX1, RUNX3, SART1, SCAMP2, SCGB3A1, SEC24C, SIN3A, SIRT6, SKP1, SKP2, SLC20A1, SLC39A1, SLC5A5, SLFN11, SLFN5, SMAD1, SMAD2, SMAD3, SMAD4, SMAD9, SMARCA4, SMARCA5, SMARCC1, SMARCC2, SMC1A, SMC3, SMCHD1, SNAI1, SNW1, SNX19, SORBS3, SPEN, SPTLC2, SQSTM1, SRRT, ST14, STAT1, STAT3, STK38, STRAP, STUB1, SUPT16H, SUPT6H, TAB1, TACC3, TBL1XR1, TCF12, TFDP1, TFDP2, TGFBR1, TGOLN2, TJP1, TLE3, TMEM108, TMEM17, TNNT2, TNPO1, TNRC6A, TNRC6B, TNRC6C, TOP2B, TP53, TPT1, TRAPPC10, TRAPPC9, TRIM25, TRIM28, TRIM3, TRIM32, TSPAN3, UBA52, UBB, UBC, USP11, USP12, USP15, USP7, VASN, VAV1, VEGFA, VPS11, VPS39, WDR11, WDR12, WDR25, WDR6, WIZ, WWC1, WWP1, WWP2, WWTR1, XIAP, XKR8, XPO5, XRCC6, YBX1, YWHAZ, YY1, ZBED4, ZBTB2, ZFP41, ZMIZ1, ZNF224, ZNF408, ZNF598, ZNF8	231
KEGG	KEGG:hsa04330	“NOTCH signaling pathway”	ADAM17, APH1A, CIR1, CREBBP, CTBP1, CTBP2, DLL1, DLL3, DLL4, DTX1, DTX2, DTX3, DTX3L, DTX4, DVL1, DVL2, DVL3, EP300, HDAC1, HDAC2, HES1, HES5, JAG1, JAG2, KAT2A, KAT2B, LFNG, MAML1, MAML2, MAML3, MFNG, NCOR2, NCSTN, NOTCH1, NOTCH2, NOTCH3, NOTCH4, NUMB, NUMBL, PSEN1, PSEN2, PSENEN, PTCRA, RBPJ, RBPJL, RFNG, SNW1	47
PID	MsigDB:M17	“NOTCH pathway”	ADAM10, ADAM12, APH1A, APH1B, CBL, CCND1, CDKN1A, CNTN1, CNTN6, CTBP1, CUL1, DLK1, DLL1, DLL3, DLL4, DNER, DNM1, DTX1, ENO1, EP300, EPS15, FBXW7, FURIN, GATA3, HDAC1, IL4, ITCH, JAG1, JAG2, KDM1A, LNX1, MAML1, MAML2, MARK2, MFAP2, MFAP5, MIB1, MYC, MYCBP, NCOR1, NCOR2, NCSTN, NEURL1, NOTCH1, NOTCH2, NOTCH3, NOTCH4, NUMB, PSEN1, PSENEN, PTCRA, RAB11A, RBBP8, RBPJ, SKP1, SKP2, SPEN, SSPO, YY1	59
MsigDB	MsigDB:M15592	NGUYEN_NOTCH1_TARGETS_UP	ABCC8, ANXA6, ARL6IP5, BRCA1, CANX, CD4, CNTN1, COL18A1, F2R, FLOT1, HDAC1, HMGCR, ITGAV, LAMB2, NQO1, PCF11, PHB2, PSMD10, PTHLH, RBP1, S100A13, SEC62, SFPQ, SLC12A3, SLC3A1, SLC4A3, STAT5A, TAGLN, TCAP	29
MsigDB	MsigDB:M14650	NGUYEN_NOTCH1_TARGETS_DN	AANAT, APEX1, ASS1, BASP1, BET1, BRD2, CADM1, CCNI, CDK11A, COL11A2, COL7A1, CRX, CYP2E1, CYR61, DAXX, DDAH2, DYNC1H1, EEF1A1, ELL2, EPHA4, ERCC5, ETS2, EXT1, F3, FNDC3A, GADD45B, GARS, GTF2I, HERPUD1, HPCAL1, HTRA2, HUWE1, IGF1R, IL1R1, ITGA2, KLF10, KLF4, KLF5, LAMA5, LIMK2, MARCKS, MEF2A, MGAT2, MTHFD2, MYC, MYO1E, NET1, NOP2, PBX3, PCDH7, PDE6A, PNN, PPIF, PPP2R1B, PSMB9, PSME4, PTGS2, PTPN13, RBMS1, REV3L, RGS2, RING1, RPE, SERP1, SERPINH1, SGSM3, SLC12A2, SLC12A4, SLC20A1, SMYD5, SRPK2, STAG2, STC2, TARS, TFAP2A, TGM3, TIPARP, TNFSF4, TP53, TP63, TRIO, TRIP12, UBR4, USP22	84
MsigDB	MsigDB:M1869	VILIMAS_NOTCH1_TARGETS_UP	BCL2A1, BIRC2, BIRC3, CARD11, CCL5, CCR7, CD2, CD28, CD3D, CD3G, CD69, CD7, CD74, CD80, CD83, CD86, CTLA4, CX3CL1, DDR1, DTX1, EGR1, EGR2, GATA3, GZMA, GZMB, HEY1, ICAM1, ID2, IL10RA, IL12B, IRF7, JUNB, LAT, LCK, NFATC1, NFKB2, NFKBIA, NOTCH3, NRARP, P2RY10, PTCRA, RAG1, RAG2, RELB, RRAS2, TARP, THY1, TNFRSF18, TOX, TRAF1, TRAT1, ZAP70	52
MsigDB	MsigDB:M1873	VILIMAS_NOTCH1_TARGETS_DN	BTK, CAMP, CCL15, CLEC10A, CMA1, CSF1R, CSF2RA, CSF3R, CTSG, FCGR2B, GFI1, GFI1B, ITGAM, ITGB2, LILRB3, LRRC25, LYZ, MATK, MPO, NCF2, NCF4, PGLYRP1, STOM, TPSAB1, TREM2, TREML1, VPREB1	27
Manually curated NOTCH targets	--	Manually curated NOTCH targets	NOTCH1, NOTCH2, NOTCH3, NOTCH4, MYC, HES1, HES2, HES4, HES5, HES6, HEY1, HEY2, HEYL, NRARP, KIT, MVP, OLFM4, CDK6, CCND1, CDKN1B, CDKN2D	21
**Total unique**				**478**

Supplemtary Table 1. List of 478 NOTCH related genes generated using pathways defined in KEGG, PID, MSigDB and PathwayCommons databases as well as manually curated NOTCH targets.
